# Downregulation of MEG3 and upregulation of *EZH2* cooperatively promote neuroblastoma progression

**DOI:** 10.1111/jcmm.17258

**Published:** 2022-03-08

**Authors:** Mujie Ye, Runnan Gao, Shiyu Chen, Meng Wei, Jing Wang, Bowen Zhang, Suwen Wu, Yuexin Xu, Peixuan Wu, Xin Chen, Jing Ma, Duan Ma, Kuiran Dong

**Affiliations:** ^1^ Department of Pediatric Surgery Children's Hospital of Fudan University National Children's Medical Center Shanghai China; ^2^ Department of Geriatric Gastroenterology Neuroendocrine Tumor Center Jiangsu Province Hospital The First Affiliated Hospital of Nanjing Medical University Institute of Neuroendocrine Tumor Nanjing Medical University Nanjing China; ^3^ Department of Biochemistry and Molecular Biology Research Center for Birth Defects Institutes of Biomedical Sciences Key Laboratory of Metabolism and Molecular Medicine Ministry of Education School of Basic Medical Sciences Fudan University Shanghai China; ^4^ Obstetrics and Gynaecology Hospital Fudan University Shanghai China; ^5^ Department of Facial Plastic and Reconstructive Surgery, Eye and ENT Hospital ENT Institute Fudan University Shanghai China

**Keywords:** epigenetics, *EZH2*, long non‐coding RNA, MEG3, neuroblastoma

## Abstract

Neuroblastoma (NB), an embryonic tumour originating from sympathetic crest cells, is the most common extracranial solid tumour type in children with poor overall prognosis. Accumulating evidence has demonstrated the involvement of long non‐coding RNA (lncRNA) in numerous biological processes and their associations with embryonic development and multiple diseases. Ectopic lncRNA expression is linked to malignant tumours. Previous studies by our team indicate that MEG3 attenuates NB autophagy through inhibition of *FOXO1* and epithelial‐mesenchymal transition via the mTOR pathway in vitro. Moreover, MEG3 and *EZH2* negatively regulate each other. In present study, we first collected 60 NB tissues and 20 adjacent tissues for Quantitative real‐time polymerase chain reaction (Q‐PCR) experiments and performed clinical correlation analysis of the results. At the same time, nude mice were used for subcutaneous tumour formation to detect the effect of MEG3 in vivo. Two NB cell lines, SK‐N‐AS and SK‐N‐BE(2)C, were overexpressed MEG3 and rescued with *EZH2* and then were subjected to proliferation, migration, invasion, apoptosis and autophagy experiments. RNA‐binding protein immunoprecipitation (RIP) and Co‐Immunoprecipitation (Co‐IP) experiments were performed to explore the molecular mechanism of MEG3 and *EZH2* interaction. Q‐PCR revealed that MEG3 expression was negatively correlated with INSS stage and risk grade of NB. Moreover, MEG3 overexpression was associated with inhibition of NB growth in vivo. MEG3 exerted an anti‐cancer effect via stimulatory effects on *EZH2* ubiquitination leading to its degradation. Conversely, *EZH2* interacted with *DNMT1* and *HDAC1* to induce silencing of MEG3. The *EZH2* inhibitor, DZNep, and *HDAC* inhibitor, SAHA, displayed synergistic activity against NB. Combined treatment with DZNep and SAHA inhibited proliferation, migration and invasion of NB through suppression of the *PI3K*/*AKT*/*mTOR*/*FOXO1* pathway. In conclusion, downregulation of MEG3 and upregulation of *EZH2* forms a feedback loop that concertedly promotes the development of NB. Combined blockage of *EZH2* and *HDAC1* with the appropriate inhibitors may therefore present an effective treatment strategy for NB cases with low MEG3 and high *EZH2* expression.

## 1 INTRODUCTION

Neuroblastoma (NB) is an embryonal tumour arising in the developing sympathetic nervous system with typical presentation in adrenal glands and/or sympathetic ganglia.^1^, ^2^ As the most common extracranial solid tumour type in children, NB is usually diagnosed in the first year of life with an average of 25–50 cases per million individuals, accounting for disproportionate morbidity and mortality among paediatric tumours.^3^, ^4^ Although the outcomes of NB have improved owing to updated and effective interventions, long‐term survival rates in children with high‐risk NB remain relatively poor.^1^, ^5^ Accumulating studies on the mechanisms underlying pathogenesis suggest that abnormalities at the genome, epigenome and transcriptome levels are involved in the occurrence of NB.^6^, ^7^, ^8^ However, our understanding of the complex pathogenic pathways underlying tumorigenesis of NB is still evolving and further research is required for effective diagnosis and identification of therapeutic targets.

With the rapid development of high‐throughput sequencing, in addition to abnormal expression of protein‐coding genes, dysregulation of non‐coding RNAs, in particular, long non‐coding RNAs (lncRNAs), has been shown to play important roles in tumour development.^9^, ^10^, ^11^ Similar to protein‐coding genes, lncRNAs possess oncogenic and tumour suppressive activities.^12^, ^13^ However, limited studies to date have investigated the involvement of lncRNAs in the development of childhood tumours, including NB.

The lncRNA, maternally expressed gene 3 (MEG3), acts as a tumour suppressor in various cancer types, including liver, lung, nasopharyngeal and stomach cancer.^14^, ^15^ Current research on MEG3 in relation to NB is mainly focused on genetic polymorphisms and genetic susceptibility. Xia and colleagues identified two MEG3 polymorphisms, rs7158663 G>A and rs4081134 G>A, in a study on 392 children with NB and 783 control subjects via the Taqman method. Stratified analysis revealed that subjects carrying the rs4081134 AG/AA genotype were susceptible to NB in subgroups older than 18 months and clinical stage III + IV. In a comprehensive investigation of MEG3 gene polymorphisms, children over 18 months of age simultaneously carrying both these risk genotypes were more likely to develop NB than those with only one or no risk genotype.^16^ However, the specific functions and mechanisms of action of MEG3 in NB remain to be established.

Polycomb repressive complex 2 (PRC2) comprises *EZH2*, *EED* and *SUZ12*, among which, *EZH2* is the only active subunit with a critical role.^17^, ^18^, ^19^ The *EZH2* catalytic SET domain catalysing histone 3 lysine 27 tri‐methylation (H3K27me3) is reported to bind and silence specific tumour suppressor genes.^20^, ^21^ Earlier, Kamijo and co‐workers demonstrated upregulation of *EZH2* in NB in association with poorer prognosis and overall survival. Furthermore, *EZH2* was shown to affect NB differentiation through regulation of *NTRK1*.^22^ Another study by Bownes et al. revealed decreased NB proliferation and in vivo tumour growth following inhibition of *EZH2*.^23^

The role of the lncRNA MEG3 in NB was previously explored by our team. Consistent with findings from other studies, our experiments supported a tumour suppressor role of MEG3, showing a negative correlation between MEG3 expression and development of NB in vitro. While our earlier results suggest that MEG3 and *EZH2* mutually regulate and jointly promote progression of NB by forming a negative feedback loop, the precise pathways remain to be clarified.^24^ The main objective of the present study was to uncover the mechanistic pathways between MEG3 and *EZH2* and explore the potential therapeutic utility of combinations of drugs targeting both genes against NB.

## 2 MATERIALS AND METHODS

### 2.1 Cell culture

The human NB cell lines, SK‐N‐BE(2)‐C and SK‐N‐AS, were kind gifts from Professor Kai Li of Children's Hospital of Fudan University. SK‐N‐BE (2)‐C and SK‐N‐AS cells were cultured in DMEM/F12 and DMEM (Biological Industries), respectively. For both cell lines, the culture medium was routinely supplemented with 10% foetal bovine serum (FBS; Gibco) and 1% penicillin‐streptomycin (New Cell & Molecular Biotech) and incubated under a humidified atmosphere with 5% CO_2_ at 37°C. Cell culture dishes were purchased from Xinyou Biotechnology Company and CELLSAVING™ from New Cell & Molecular Biotech.

### 2.2 Quantitative real‐time polymerase chain reaction

Sixty NB tissues and twenty control tissues were obtained from patients subjected to surgery at Children's Hospital of Fudan University. Informed consent was acquired from every patient. Total RNA was isolated from the above tissues or cell lines using TRIzol reagent (Takara) according to the manufacturer's instructions. cDNA was generated from reverse‐transcribed RNA using a specific reverse transcription kit, in keeping with the manufacturer's protocol. Quantitative real‐time polymerase chain reaction (Q‐PCR) was performed using Hieff UNICON^®^ Universal Blue qPCR SYBR Green Master Mix (Yeasen) with a Roche instrument for determination of relative RNA levels. Results were calculated using the ΔΔ*C*_t_ method and normalized to the reference housekeeping gene GAPDH. The primers used were as follows: MEG3‐forward, 5′‐CCTCACCTCCAATTTCCTCTTC‐3′, MEG3‐reverse, 5′‐TCCAGCAGCTAACCTCATTAAC‐3′; GAPDH‐forward 5′‐GGAGCGAGATCCCTCCAAAAT‐3′, GAPDH‐reverse, 5′‐GGCTGTTGTCATACTTCTCATGG‐3′. Final data were analysed with GraphPad Prism 5 software.

### 2.3 Animal assays

Control and overexpression MEG3 SK‐N‐AS cells were cultured and washed three times using PBS, resuspended, and then counted in PBS. The cell density was adjusted to 5 × 10^7^ cells/ml. Twelve nude mice (BALB/c) were randomly divided into two groups and subcutaneously inoculated with 5 × 10^6^ cells (100 μl cell suspension), then tumour volume was recorded once a week. After 4 weeks, mice were sacrificed, the tumours were taken out, photographed, weighed and fixed in formalin solution (only 50% of mice successfully grow tumours). The animal assays were approved by the Ethics Committee of Children's Hospital of Fudan University.

### 2.4 Western blot

Total proteins were extracted using NP40 lysis buffer (Beyotime) containing PMSF (Beyotime) to protect against degradation of protein. Proteins were quantified using the Bradford assay with the Coomassie Brilliant Blue G250 reagent kit (Beyotime). After denaturation of the extracted protein by boiling at 95°C for 10 min, equal amounts of all protein samples were separated via SDS‐PAGE (New Cell & Molecular Biotech) according to molecular weight of target protein and transferred to membranes. Next, membranes were blocked with 8% skimmed milk for 1 h and incubated overnight with corresponding primary antibodies at 4°C. On day 2, all reactions were operated at room temperature. After washing with TBST three times, membranes were incubated with HRP‐conjugated secondary antibodies for 1 h. Immunoblot signals were obtained from an imaging system using an Enhanced Chemiluminescent Reagent kit (New Cell & Molecular Biotech). GAPDH or β‐actin was selected as the loading control. All the antibodies used in this study are listed in Table S1.

### 2.5 Immunohistochemical analysis

Human NB tumour and paired control tissues were collected from the Children's Hospital of Fudan University. Samples were soaked, embedded, dewaxed and incubated in citric acid antigen retrieval buffer, followed by 3% BSA for blocking. Slides were incubated overnight at 4°C with primary antibodies specific for Ki67 (1:200; Servicebio) and EZH2 (1:50, CST). On day 2, slides were washed three times and incubated with secondary antibody (1:200; Servicebio) for 1 h at room temperature. DAB colour developing solution was added, followed by haematoxylin staining. Finally, slides were dehydrated and mounted and images were obtained via microscopy (Thermo).

### 2.6 Cell proliferation and colony formation

Cell proliferation ability was measured using Cell Counting Kit‐8 (CCK‐8; Yeasen). SK‐N‐AS and SK‐N‐BE(2)C cells were seeded into 96‐well plates at a density of 2 × 10^3^ cells and 4 × 10^3^ cells per well, respectively. CCK‐8 (10 μl) was added to cells and incubated for 2 h at 37°C, and absorbance was measured at 450 nm daily for 4 consecutive days. A 5‐ethynyl‐20‐deoxyuridine (EdU) assay kit (Ribobio) was utilized to determine cell proliferation ability. Cells were seeded into 96‐well plates at a density of 1 × 10^5^ cells each well, incubated in 50 μM EdU buffer for 2 h at 37°C, fixed with 4% formaldehyde for 0.5 h and permeabilized with 0.1% Triton X‐100 for 20 min. Next, EdU solution was added to cultures, followed by staining of nuclei with Hoechst 33342, and results were visualized under a fluorescence microscope (Thermo). For the colony formation assay, 1.5 × 10^3^ SK‐N‐AS and 3 × 10^3^ SK‐N‐BE(2)C cells per well were seeded into 6‐well plates and cultured in the appropriate medium for about 2 weeks. After 14 days of incubation, plates were washed twice with PBS, fixed in 4% paraformaldehyde (PFA) for 15–20 min and stained with 0.1% crystal violet solution for 10–15 min for further analysis.

### 2.7 Migration and invasion assays

Migration and invasion assays were performed using 24‐well plates containing 8 μm pore size transwell filter inserts with or without pre‐coated diluted matrigel (1:5; Becton Dickinson). SK‐N‐AS cells at a density of 1 × 10^5^ (migration) and 2 × 10^5^ (invasion) and SK‐N‐BE (2)C cells at a density of 2 × 10^5^ (migration) and 4 × 10^5^ (invasion) diluted in serum‐free medium were placed in the upper chamber and medium containing 30% FBS added to the lower chamber. After incubation for 48 h at 37°C, cells on the underside of the membrane were fixed with 4% PFA for 15 min and stained with 0.1% crystal violet solution within 20 min for further analysis. Penetrating cells from five random fields were counted under the microscope.

### 2.8 Apoptosis detection

Cells were collected in a 6 cm culture dish, washed twice with PBS and digested with trypsin without EDTA. Next, 5 μl PE and 7‐amino actinomycin D staining solution were added after fixation for 15 min in the dark. Samples were subjected to flow cytometry and analysed with FlowJo software.

### 2.9 Plasmid construction

All short hairpin RNAs were designed using the website of Sigma. The target sequences of genes were as follows: EZH2, 5′‐CCCAACATAGATGGACCAAAT‐3′; UCHL1, 5′‐CGGGTAGATGACAAGGTGAAT‐3′; DNMT1, 5′‐GCCCAATGAGACTGACATCAA‐3′; DNMT3A, 5′‐CCACCAGAAGAAGAGAAGAAT‐3′; DNMT3B, GCCTCAAGACAAATTGCTATA‐3′; HDAC1, 5′‐GCTGCTCAACTATGGTCTCTA‐3′; HDAC2, 5′‐GCAAATACTATGCTGTCAATT‐3′. EZH2 and ΔSET EZH2 plasmids were synthesized by Shanghai Generay Biotech Co., Ltd. DNMT1 and HDAC1 overexpression plasmids were purchased from Shanghai Genomeditech and Shandong WZ Biotech Co., Ltd, respectively.

### 2.10 RNA‐binding protein immunoprecipitation

Cell pellets cultured in a 10 cm dish were collected, and the same volume of RNA‐binding protein immunoprecipitation (RIP) lysis buffer was added to the tube. Samples were incubated overnight at −80°C after splitting on ice for 5 min. Next, protein A/G magnetic beads (Thermo) were washed five times with NT2 buffer and incubated with 5 μg antibody. After 2 h, samples were re‐washed with NT2 buffer and mixed with supernatant fractions of the lysates overnight at 4℃. The next day, supernatants were washed five times with NT2 buffer and proteinase K buffer added for 30 min at 55℃. RNA was extracted with TRIzol reagent, and reverse transcription and Q‐PCR were performed as described above.

### 2.11 Co‐immunoprecipitation experiments

NP40 lysates (1 ml) were added to 10 cm dish cells and proteins extracted using the conventional WB method. After incubation with 2 µg antibody for 2 h at 4°C, 30 µl protein A/G agarose beads (Santa Cruz) was added and inverted overnight. The next day, samples were washed thoroughly with NP40 lysis buffer (Regal Biology) five times, incubated in 30 µl of 2×SDS‐PAGE sample loading buffer (Yeasen) and boiled at 95°C for 5 min for subsequent WB experiments.

### 2.12 ChIP‐seq and RNA‐seq

For ChIP‐seq, cells in a 10 cm dish were resuspended in 10 ml medium. Next, 270 μl of 37% formaldehyde solution was added at room temperature for 10 min, followed by 540 μl of 2.5 mol glycine for 5 min. ChIP lysis buffer (1.5 ml) was added to samples on ice for 15–20 min, and DNA fragments were generated with a sonicator (200–1000 bp in size). Next, 10 μg antibody was added to samples and rotated overnight at 4°C. The next day, 50 μl magnetic beads were added and reacted for 4 h at 4°C. After thorough washing, DNA was eluted and sequenced following library construction. For RNA‐seq experiments, RNA was extracted from a plate of 10 cm cells per sample with TRIzol and the cDNA library constructed. GO and KEGG analysis were performed after sequencing.

### 2.13 Statistical analysis

Every assay was repeated independently at least three times. Results were presented as the mean ± SD. Groups were compared using t‐test, and *p* < 0.05 was considered statistically significant.

## 3 RESULTS

### 3.1 MEG3 inhibits NB growth in vivo and *EZH2* is highly expressed in NB

**FIGURE 1:**
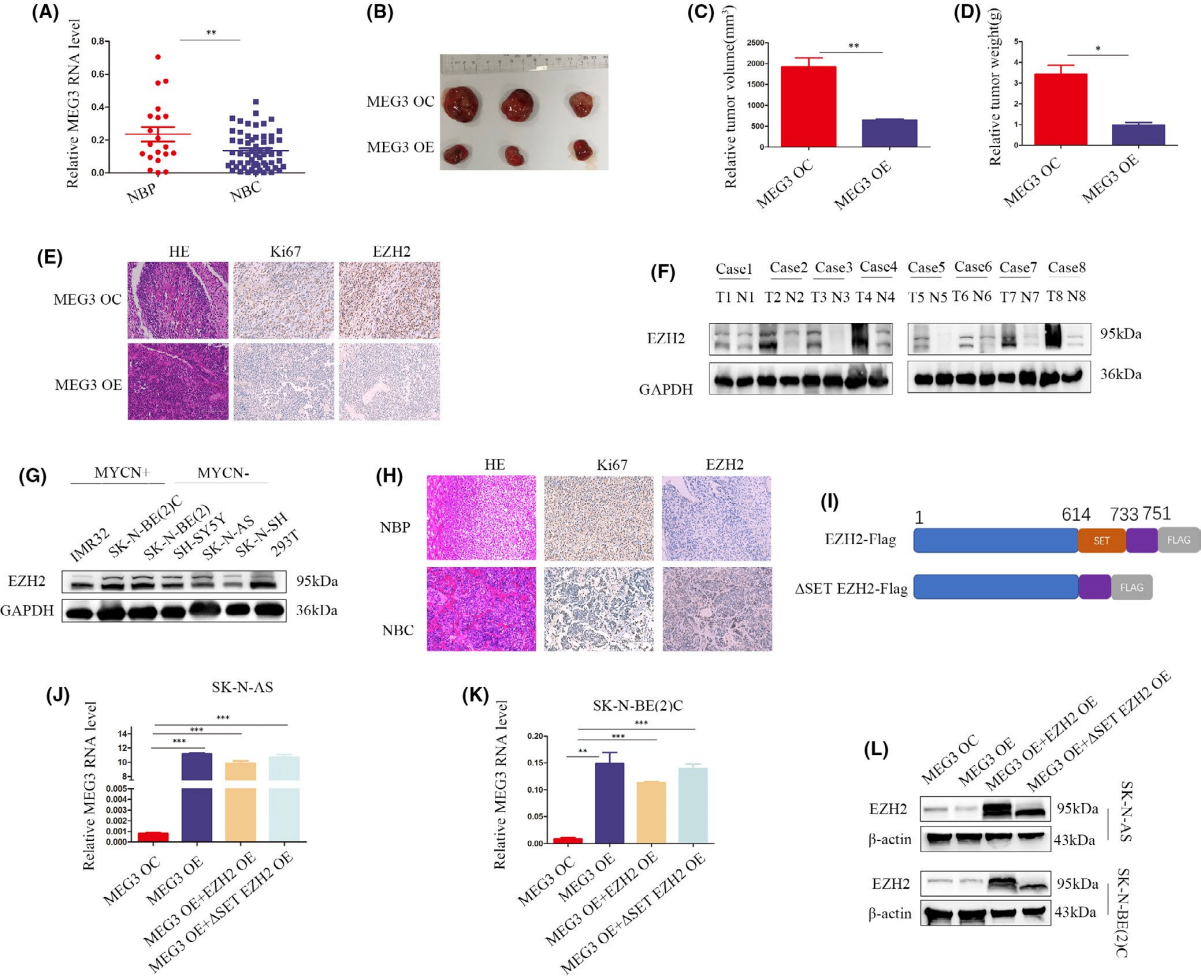
MEG3 inhibits NB growth *in vivo* and EZH2 is highly expressed in NB. A, Quantitative real‐time polymerase chain reaction (Q‐PCR) analysis of MEG3 expression in NB (NBC, n = 60) and adjacent normal adrenal tissues (NBP, *n* = 20); B, General view of MEG3 overexpression (MEG3 OE) and control groups (MEG3 OC) in nude mice; C, D, Tumour volume and weight of nude mice in MEG3 upregulation and control groups; E, Haematoxylin‐eosin staining. Ki67 and EZH2 immunohistochemistry in subcutaneous tumours of MEG3 overexpression and control groups; F, Representative Western blot of EZH2 in NB and adjacent normal adrenal tissues (*n* = 8), T signifies tumour and N normal; G, EZH2 protein expression in MYCN amplification(IMR32, SK‐N‐BE(2)C and SK‐N‐BE(2)) and non‐amplification NB cell lines(SH‐SY5Y, SK‐N‐AS and SK‐N‐NH); H, Representative haematoxylin‐eosin staining, Ki67 and EZH2 immunohistochemistry in NB and adjacent normal adrenal tissues; I, Pattern diagram of EZH2 and deletion SET domain EZH2 (ΔSET EZH2); J, K, Q‐PCR analysis of MEG3 in control, MEG3 overexpression, EZH2 rescue and ΔSET EZH2 rescue groups in SK‐N‐AS and SK‐N‐BE(2)C cell lines; L, Western blot of MEG3 expression in control, MEG3 overexpression, EZH2 rescue and ΔSET EZH2 rescue groups in SK‐N‐AS and SK‐N‐BE(2)C cell lines (*p < 0.05, **p < 0.01, ***p < 0.001)

Previous experiments by our team support anti‐tumour activity of MEG3 in NB. The present study was conducted on an expanded clinical sample size. Q‐PCR analyses showed significant downregulation of MEG3 in NB compared to adjacent adrenal tissues (Figure 1A). In clinical correlation analysis, MEG3 was negatively correlated with INSS stage and risk grade of NB (Table 1, Table S2). Moreover, animal experiments revealed an association of MEG3 overexpression with NB growth inhibition in vivo (Figure 1B–D). Immunohistochemical analyses further disclosed that upregulation of MEG3 induces a decrease in *Ki67* and *EZH2* levels in vivo (Figure 1E).

**TABLE 1:** Correlation analysis of MEG3 expression and clinical characteristics

Variables	MEG3 expression		*p* value
	High (*n *= 30)	Low (*n* = 30)	
Gender			
Male	15	15	0.602
Female	15	15	
Age			
≥18 months	22	23	0.500
<18 months	8	7	
INSS stage			
I/II/4s	11	5	0.045
III/IV	14	22	
Unknown	5	3	
Risk degree			
Low/intermediate	15	6	0.027
High	13	19	
Unknown	2	5	
MYCN state			
No amplification	18	12	0.161
Amplification	4	7	
Unknown	8	11	
Metastasis			
Yes	17	19	0.396
No	13	11	

To validate the expression of *EZH2* in NB, tumour tissues and adjacent adrenal tissues were examined via Western blot and immunohistochemistry. Our results showed significantly higher levels of *EZH2* in NB compared to adjacent normal tissues (Figure 1F,H). Moreover, higher expression of *EZH2* was observed in *MYCN* amplified NB cells than *MYCN* non‐amplified cells (Figure 1G). To ascertain whether *EZH2* could rescue the tumour suppressor effect of MEG3, *EZH2* or *EZH2* depleted of the SET domain was overexpressed in cells stably transfected with MEG3 (Figure 1I). Q‐PCR and Western blot data validated the successful construction of both SK‐N‐AS and SK‐N‐BE (2) C cells (Figure 1J–L).

### 3.2 Upregulation of *EZH2* rescues the tumour inhibitory effects of MEG3

**FIGURE 2:**
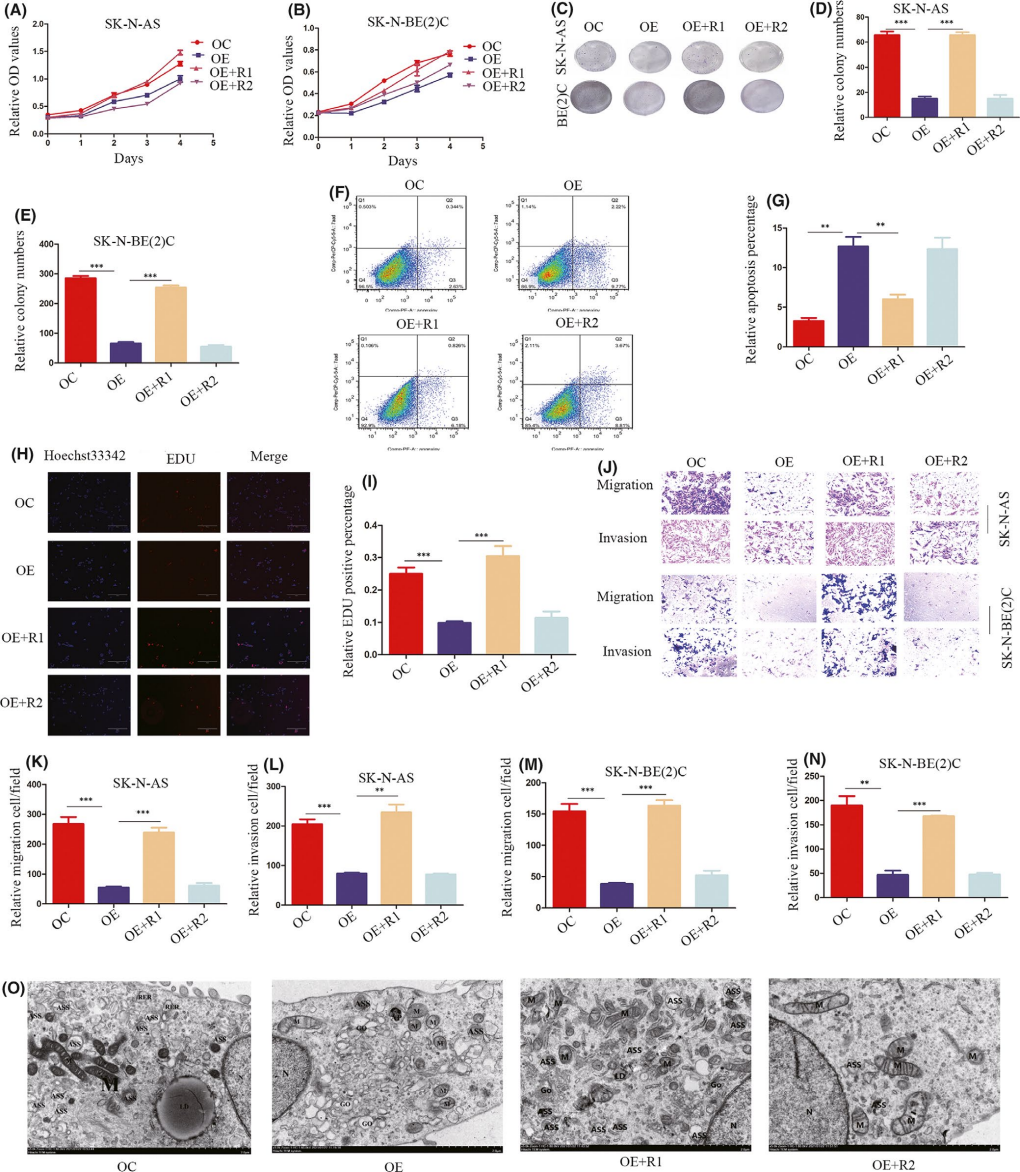
EZH2 promotes cell proliferation, colony formation, migration, invasion and autophagy while inhibiting apoptosis. A, B, CCK‐8 analysis in control (MEG3 OC), MEG3 overexpression (MEG3 OE), EZH2 rescue (OE + R1) and ΔSET EZH2 rescue (OE+R2) groups of SK‐N‐AS and SK‐N‐BE(2)C cell lines; C, D, E, Colony formation assay in control, MEG3 overexpression, EZH2 rescue and ΔSET EZH2 rescue groups of SK‐N‐AS and SK‐N‐BE(2)C cell lines; F, G, Apoptosis detection via flow cytometry in control, MEG3 overexpression, EZH2 rescue and ΔSET EZH2 rescue groups of SK‐N‐AS and SK‐N‐BE(2)C cell lines; H, I, EdU assay in control, MEG3 overexpression, EZH2 rescue and ΔSET EZH2 rescue groups of SK‐N‐AS and SK‐N‐BE(2)C cell lines; J–N, Transwell assay in control, MEG3 overexpression, EZH2 rescue and ΔSET EZH2 rescue groups of SK‐N‐AS and SK‐N‐BE(2)C cell lines; O, Electron microscopy in control, MEG3 overexpression, EZH2 rescue and ΔSET EZH2 rescue groups of SK‐N‐AS and SK‐N‐BE(2)C cell lines (scale, 2 µm). ASS represents autolysosome, AP autophagosome, M mitochondria, N nucleus, Go golgi, LD lipid droplets, RER rough endoplasmic reticulum, R1 EZH2 rescue and R2 ΔSET EZH2 rescue (**p < 0.01, ***p < 0.001)

MEG3 has been shown to inhibit NB cell proliferation, migration, invasion and autophagy and promote apoptosis in our previous studies.^24^ In the CCK‐8 assay, wild‐type *EZH2*, but not *EZH2* depleted of the SET domain, facilitated cell proliferation (Figure 2A,B). Similarly, *EZH2*, but not ΔSET *EZH2*, rescued colony formation ability (Figure 2C–E). Flow cytometry showed that apoptosis induced by overexpression of MEG3 could be rescued by *EZH2*, but not ΔSET *EZH2* (Figure 2F,G; Figure S1A,B). In accordance with CCK‐8 findings, EdU results validated the proliferative effect of wild‐type *EZH2* and the SET domain in NB cells (Figure 2H,I). To confirm these findings, we examined the effects of *EZH2* and ΔSET *EZH2* on NB cell metastasis. Consistent with the above results, *EZH2*, but not ΔSET *EZH2*, promoted migration and invasion of NB cells (Figure 2J–N). In addition, electron microscopy results indicated that autophagy inhibited by MEG3 is increased by ectopic *EZH2* and that depletion of SET leads to loss of this function (Figure 2O). Therefore, we propose that MEG3 exerts anti‐cancer activity through negatively regulating *EZH2* in NB cells. Furthermore, the SET domain appears indispensable for *EZH2* to exert its oncogenic effects.

### 3.3 *EZH2* promotes *FOXO1*‐mediated autophagy and *mTOR* mediates epithelial‐mesenchymal transition

**FIGURE 3:**
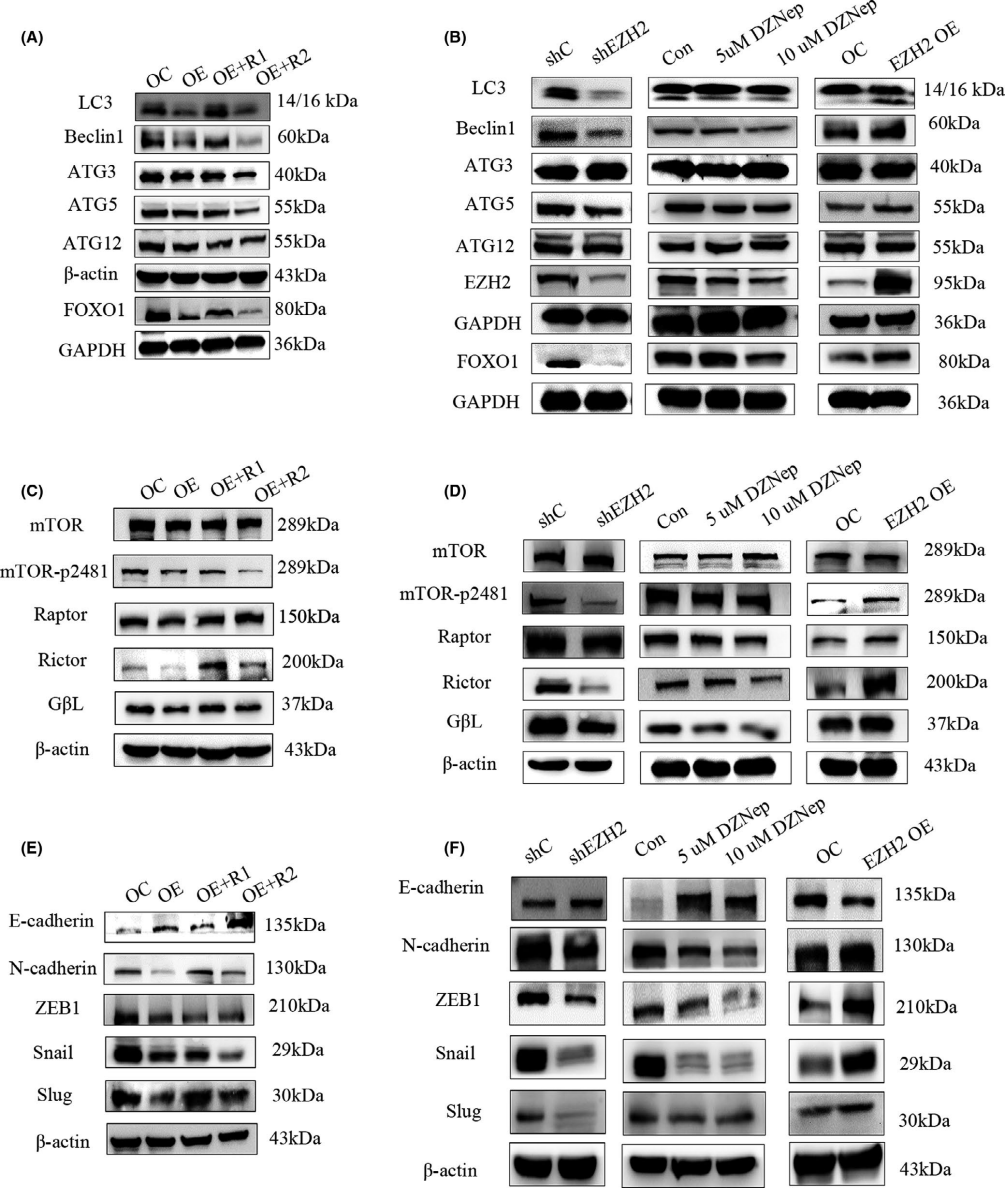
EZH2 activates autophagy, mTOR and EMT inhibited by MEG3. A, Autophagy marker detection in control, MEG3 overexpression, EZH2 rescue and ΔSET EZH2 rescue groups of SK‐N‐AS cells; B, Autophagy marker detection in EZH2 knockdown, 48 h EZH2 inhibitor DZNep (5 μm and 10 µm) treatment and EZH2 overexpression groups compared to control; C, mTOR signalling pathway protein detection in control, MEG3 overexpression, EZH2 rescue and ΔSET EZH2 rescue groups of SK‐N‐AS cells; D, mTOR signalling pathway protein detection in EZH2 knockdown, 48 h EZH2 inhibitor DZNep (5 μm and 10 μm) treatment and EZH2 overexpression groups compared with control; E, EMT marker detection in control, MEG3 overexpression, EZH2 rescue and ΔSET EZH2 rescue groups of SK‐N‐AS cells; F, EMT marker detection in EZH2 knockdown, 48 h EZH2 inhibitor DZNep (5 μm and 10 μm) treatment and EZH2 overexpression groups compared with control

In addition to cell phenotype, we explored whether the signalling pathway inhibited by MEG3 could be reactivated by *EZH2*. Western blot experiments showed that *EZH2*, but not ΔSET *EZH2*, could reactivate *FOXO1*‐mediated autophagy and *mTOR*‐induced EMT (Figure 3A,C,E). However, *EZH2* exerted no effect on the autophagy markers *ATG3* and *ATG12*. Next, *EZH2* was depleted via short hairpin RNA, which led to suppression of both autophagy and EMT. Notably, *FOXO1* and *mTOR* pathways were also inhibited upon downregulation of *EZH2*. In accordance with data from *EZH2* silencing experiments, DZNep, an *EZH2* inhibitor, induced significant suppression of *FOXO1* and *mTOR* pathways and decreased that of autophagy and EMT markers. Conversely, upregulation of *EZH2* in NB cells increased autophagy and EMT markers and activated the *FOXO1* and *mTOR* pathways (Figure 3B,D,F). The collective findings demonstrate that MEG3‐mediated inhibition of autophagy and EMT through *FOXO1* and *mTOR* is achieved via *EZH2*, in particular, the SET domain.

### 3.4 RNA‐seq and ChIP‐seq experiments show association of MEG3 and *EZH2* with the *PI3K*/*AKT* pathway

**FIGURE 4:**
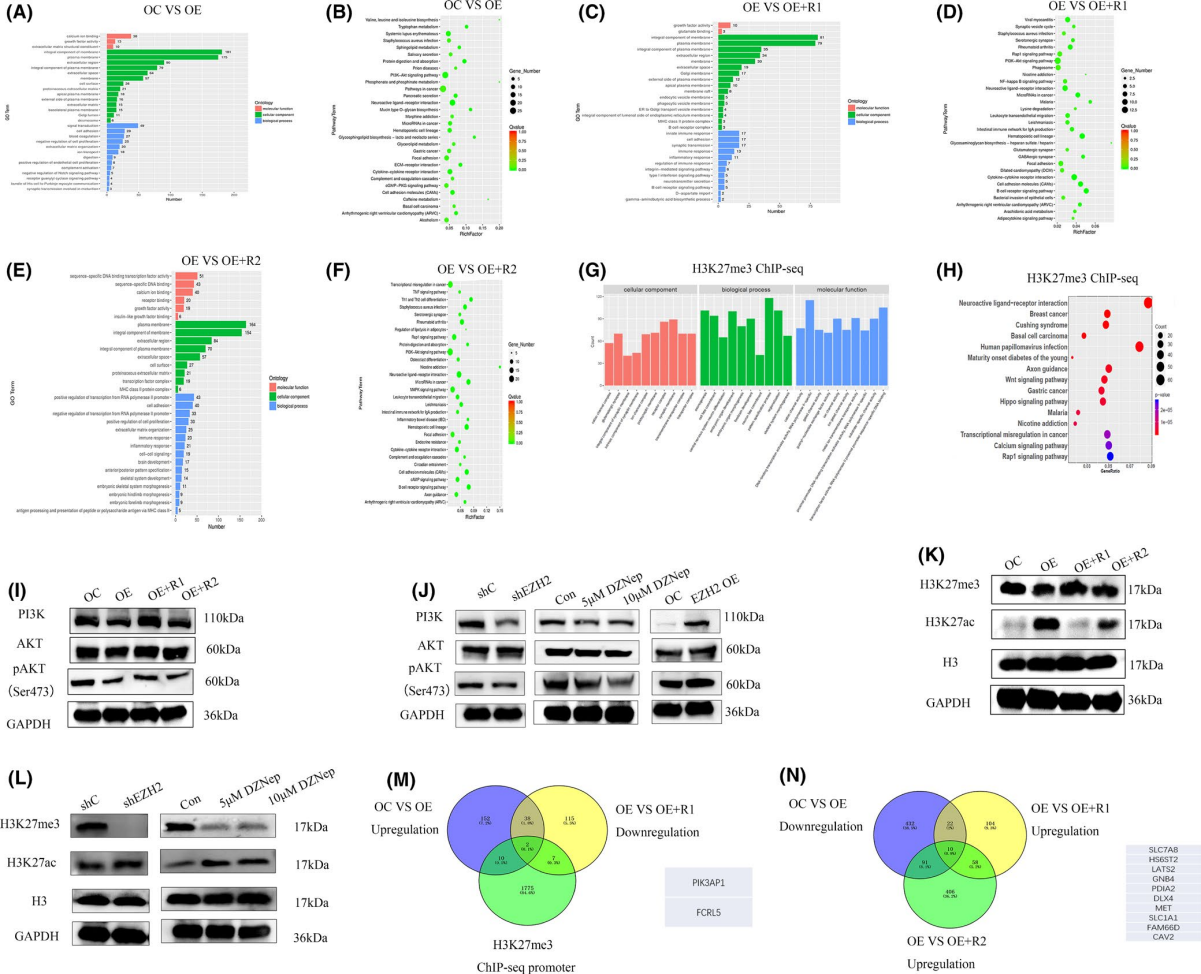
MEG3 inhibits the PI3K/AKT pathway via EZH2. A, B, GO and KEGG analyses of RNA‐seq data in MEG3 overexpression and control groups; C, D, GO and KEGG analyses of RNA‐seq data in MEG3 overexpression and EZH2 rescue groups; E, F, GO and KEGG analysis of RNA‐seq data in MEG3 overexpression and ΔSET EZH2 rescue groups; G, H, GO and KEGG analyses of ChIP‐seq data in H3K27me3; I, PI3K/AKT detection in control, MEG3 overexpression, EZH2 rescue, and ΔSET EZH2 rescue groups of SK‐N‐AS cells; J, PI3K/AKT detection in EZH2 knockdown, 48 h EZH2 inhibitor DZNep (5 μm and 10 μm) treatment, and EZH2 overexpression groups compared with control; K, Detection of histone H3 modifications in control, MEG3 overexpression, EZH2 rescue and ΔSET EZH2 rescue groups of SK‐N‐AS cells; L, Detection of histone H3 modifications in EZH2 knockdown and 48 h EZH2 inhibitor DZNep (5 μm and 10 μm) treatment groups; M, Genes upregulated by MEG3, downregulated after EZH2 rescue and enriched in the promoter region of H3K27me3 ChIP‐seq are crossed; N, Genes downregulated after overexpression of MEG3, upregulated after EZH2 rescue and upregulated after ΔSET EZH2 rescue are crossed

To further identify the potential downstream molecules, RNA‐seq and ChIP‐seq were performed. In MEG3 overexpression and control groups, 757 genes were differentially expressed, including 202 upregulated and 555 downregulated genes. Between *EZH2*‐ rescue and control groups, 162 genes were downregulated and 194 were upregulated (356 differently expressed genes in total). Among ΔSET *EZH2* rescue and control groups, 683 genes were differentially expressed, including 565 upregulated and 118 downregulated genes. GO and KEGG analyses revealed a potential association of *EZH2* with the *PI3K*/*AKT* pathway (Figure 4A–F). In addition, H3K27me3 ChIP‐seq showed an association of *EZH2* with several biological processes and signalling pathways, such as the *Wnt*, *Hippo* and *PI3K*/*AKT* (not ranked within the top 15 pathways) (Figure 4G,H). Western blot data suggested that MEG3 inhibited the *PI3K*/*AKT* pathway, which could be reactivated by *EZH2* but not ΔSET *EZH2* (Figure 4I,J). The histone methyltransferase, *EZH2*, promoted H3K27me3 and reduced H3K27ac expression through activity of the SET domain (Figure 4K). Conversely, inhibition of *EZH2* led to H3K27me3 suppression and induction of H3K27ac (Figure 4L). To identify the genes dependent on and independent of PRC2, RNA‐seq and ChIP‐seq were conducted for detection of potential downstream molecules (Figure 4M,N).

### 3.5 *UCHL1* serves as a bridge mediating regulation of *EZH2* by MEG3

**FIGURE 5:**
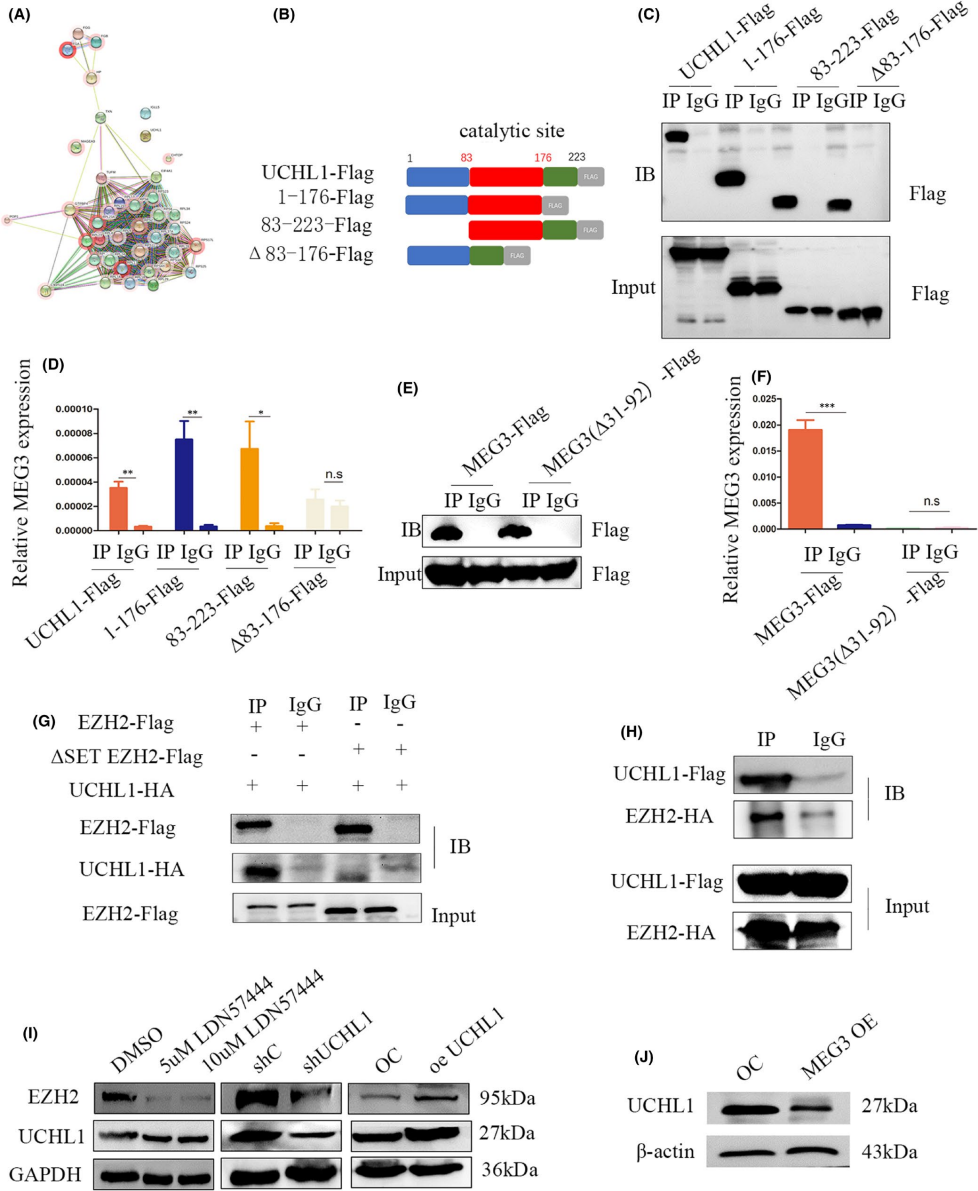
UCHL1 acts as a bridge interacting with both MEG3 and EZH2. A, PPI (protein‐protein interaction) of enriched proteins via MEG3 CHIRP; B, UCHL1‐truncated body pattern diagram; C, Western blot of UCHL1‐truncated body RIP assay; D, RIP‐QPCR analysis of MEG3 and UCHL1‐truncated body; E, Western blot of MEG3 (full‐length) and MEG3 (31–92 bp deletion) RIP assay; F, RIP‐QPCR analysis for MEG3 and MEG3 truncated body by UCHL1; G, H, Co‐IP of EZH2 and UCHL1; I, Western blot for EZH2 with UCHL1 knockdown, LDN57444 (treatment with 5 or 10 μm UCHL1 inhibitor for 48 h) and UCHL1 overexpression; J, Western blot for UCHL1 in MEG3 overexpression and control cells (*p < 0.05, **p < 0.01, ***p < 0.001)

Previously, we demonstrated that MEG3 promotes degradation of *EZH2* via ubiquitination and interacts with the deubiquitinase *UCHL1*, based on CHIRP experiments (Figure 5A). *UCHL1* is a thiol protease containing a total of 223 amino acids (among which aa 83–176 comprise the catalytic site) that hydrolyses a peptide bond at the C‐terminal glycine of ubiquitin. Using information from the database, we speculated that MEG3 (31–92 bp) binds *UCHL1* at the 83–176 aa region (Figure S1C). To examine this theory, truncation and deletion mutants of *UCHL1* were generated (Figure 5B). RIP experiments confirmed interactions between the domain comprising 83–176 aa of *UCHL1* and MEG3 (Figure 5C,D). Notably, MEG3 lacking 31–92 bp lost the ability to bind *UCHL1* (Figure 5E,F). Data from the Co‐IP assay suggested that *UCHL1* may bind the SET domain of *EZH2* (Figure 5G,H) and immunofluorescence experiments revealed co‐localization of *EZH2* and *UCHL1* in NB cells (Figure S1D). Downregulation of *UCHL1* via short hairpin RNA or its inhibitor, LDN57444, induced a decrease in *EZH2* and, conversely, its upregulation led to increased *EZH2* expression (Figure 5I). Furthermore, overexpression of MEG3 was associated with reduced levels of *UCHL1* (Figure 5J). Based on the collective findings, we propose that MEG3 interacts with *UCHL1* and inhibits its expression, thus suppressing the *EZH2* level by promoting its degradation via ubiquitination.

### 3.6 *EZH2*, *DNMT1* and *HDAC1* collectively induce silencing of MEG3 in NB

**FIGURE 6:**
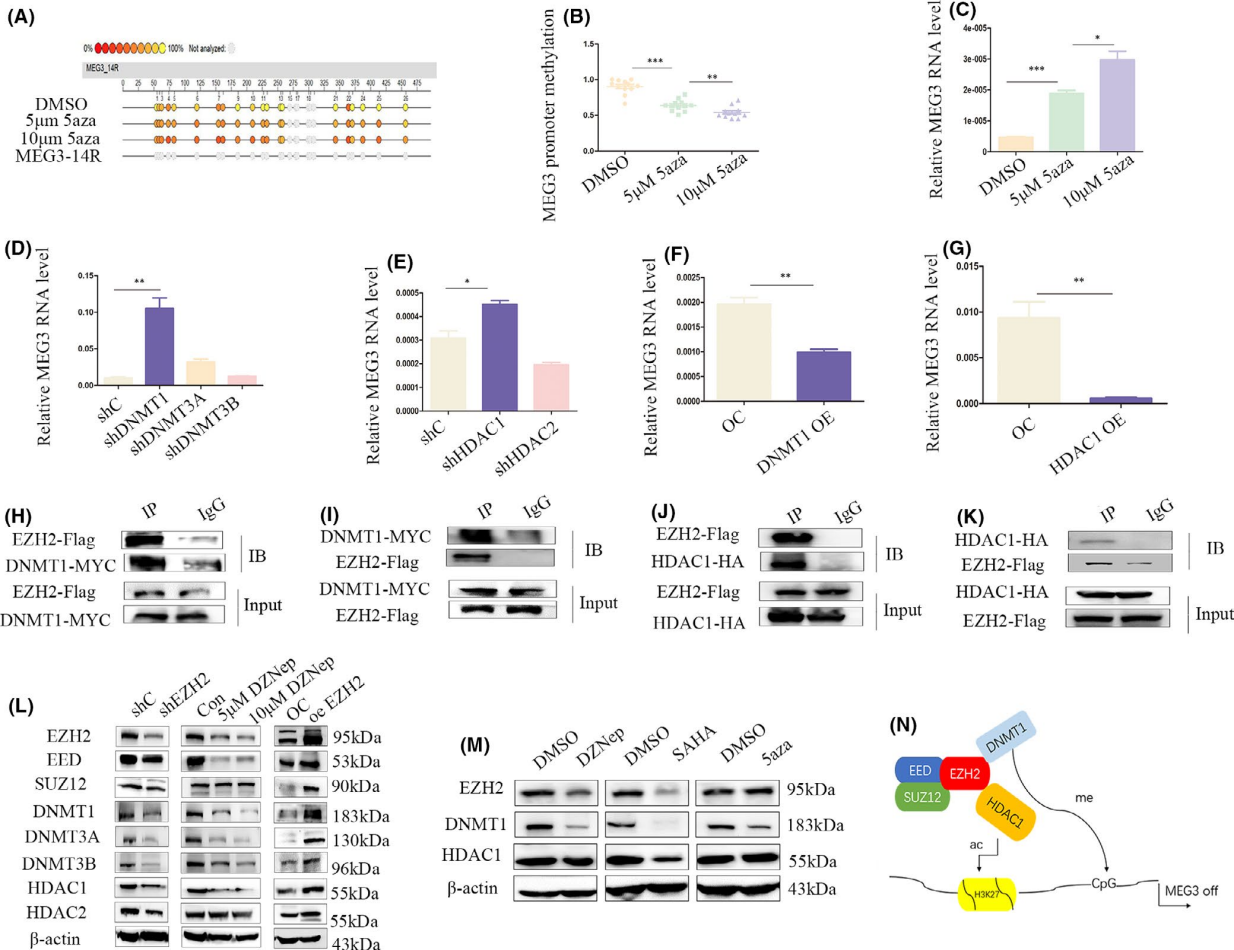
EZH2 interacts with DNMT1 and HDAC1 to induce MEG3 silencing. A, B, Methylation of MEG3 promoter treated with 5 μm and 10 μm 5‐Aza for 48 h; C, Quantitative real‐time polymerase chain reaction (Q‐PCR) analysis of MEG3 in DMSO, 5 μm 5‐Aza and 10 μm 5‐Aza groups; D, Q‐PCR analysis of MEG3 in DNMT1, DNMT3A and DNMT3B knockdown groups; E, Q‐PCR analysis of MEG3 in HDAC1 and HDAC2 knockdown groups; F, G, Q‐PCR analysis of MEG3 expression in DNMT1 and HDAC1 overexpression groups; H, I, Co‐IP for EZH2 and DNMT1; J, K, Co‐IP for EZH2 and HDAC1; L, Western blot for the PRC2 complex, DNMT and HDAC in EZH2 knockdown, 48 h EZH2 inhibitor DZNep (5 and 10 μm) treatment and EZH2 overexpression groups compared with control; M, Western blot for EZH2, DNMT1 and HDAC1 in DZNep, SAHA and 5‐Aza treatment groups; N, Schematic diagram of the upstream regulatory molecular pathway of MEG3 (*p < 0.05, **p < 0.01, ***p < 0.001)

Several CpG islands in the MEG3 promoter region are hypermethylated, inducing its downregulation. DNA methylation sequencing revealed that 5‐Aza inhibits the methylation level of the MEG3 promoter, with 10 μm 5‐Aza exerting a stronger inhibitory effect than 5 μm 5‐Aza (Figure 6A,B). Moreover, 5‐Aza promoted MEG3 expression through suppression of promoter methylation (Figure 6C). Data from DNA methyltransferase (*DNMT*) knockdown experiments (including *DNMT1*, *DNMT3A*, and *DNMT3B*; Figure S1G,J,K,L) showed that *DNMT1* exerts a negative regulatory effect on MEG3 (Figure 6D). Similarly, *HDAC1* and *HDAC2* silencing experiments were performed (Figure S1H,I,M,N). Downregulation of *HDAC1* induced a significant increase in MEG3 expression (Figure 6E). Conversely, upregulation of *DNMT1* and *HDAC1* resulted in suppression of MEG3 (Figure 6F,G; Figure S1O,P). In view of the known interactions between epigenetic factors and our earlier finding that *EZH2* negatively regulates MEG3 expression, interactions of *EZH2* with *DNMT1* and *HDAC1* were further validated in this study (Figure 6H,I,J,K). Furthermore, downregulation of *EZH2* with short hairpin RNA or DZNep led to inhibition of the PRC2 complex, *DNMT* and *HDAC* molecules (Figure 6L). Following treatment of NB cells with 5 µm or 10 µm DZNep, SAHA and 5‐Aza, DZNep and SAHA were shown to exert a synergistic effect while 5‐Aza specifically suppressed the expression of *DNMT1*, but not *EZH2* or *HDAC1*. The inhibitory effect on *DNMT1* could be replaced by DZNep and SAHA (Figure 6M). Our collective results suggest that *EZH2*, *DNMT1* and *HDAC1* form a complex that inhibits MEG3 expression (Figure 6N).

### 3.7 Combined treatment with DZNep and SAHA inhibits the malignant biological behaviour of NB through the *PI3K*/*AKT*/*mTOR*/*FOXO1* pathway

**FIGURE 7:**
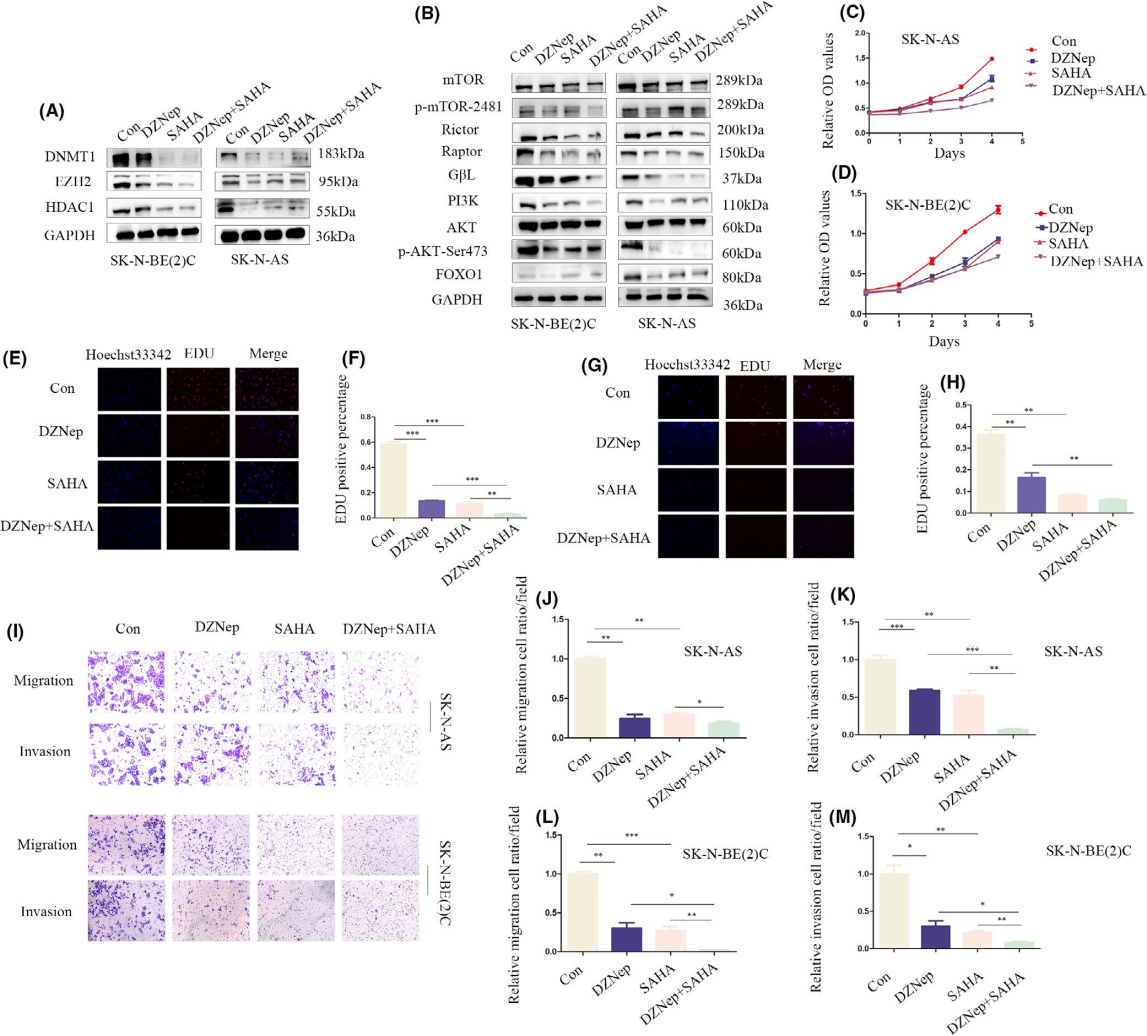
Combination of DZNep and SAHA inhibits NB proliferation, migration and invasion through the PI3K/AKT/mTOR/FOXO1 pathway. A, Western blot for EZH2, DNMT1 and HDAC1 in control, DZNep, SAHA and DZNep + SAHA groups; B, Western blot for PI3K/AKT/mTOR/FOXO1 pathway components in control, DZNep, SAHA and DZNep + SAHA groups; C, D, CCK‐8 assay of control, DZNep, SAHA and DZNep + SAHA groups of SK‐N‐AS and SK‐N‐BE(2)C cells; E, F, EdU assay for control, DZNep, SAHA and DZNep + SAHA groups of SK‐N‐AS cells; G, H, EdU assay for control, DZNep, SAHA and DZNep + SAHA groups of SK‐N‐BE(2)C cells; I–M, Transwell assay for control, DZNep, SAHA and DZNep + SAHA groups of SK‐N‐AS and SK‐N‐BE(2)C cells, the magnification is 100 times. All cells were treated with 10 μm DZNep and 10 μm SAHA for 48 h. *p < 0.05, **p < 0.01, ***p < 0.001

**FIGURE 8:**
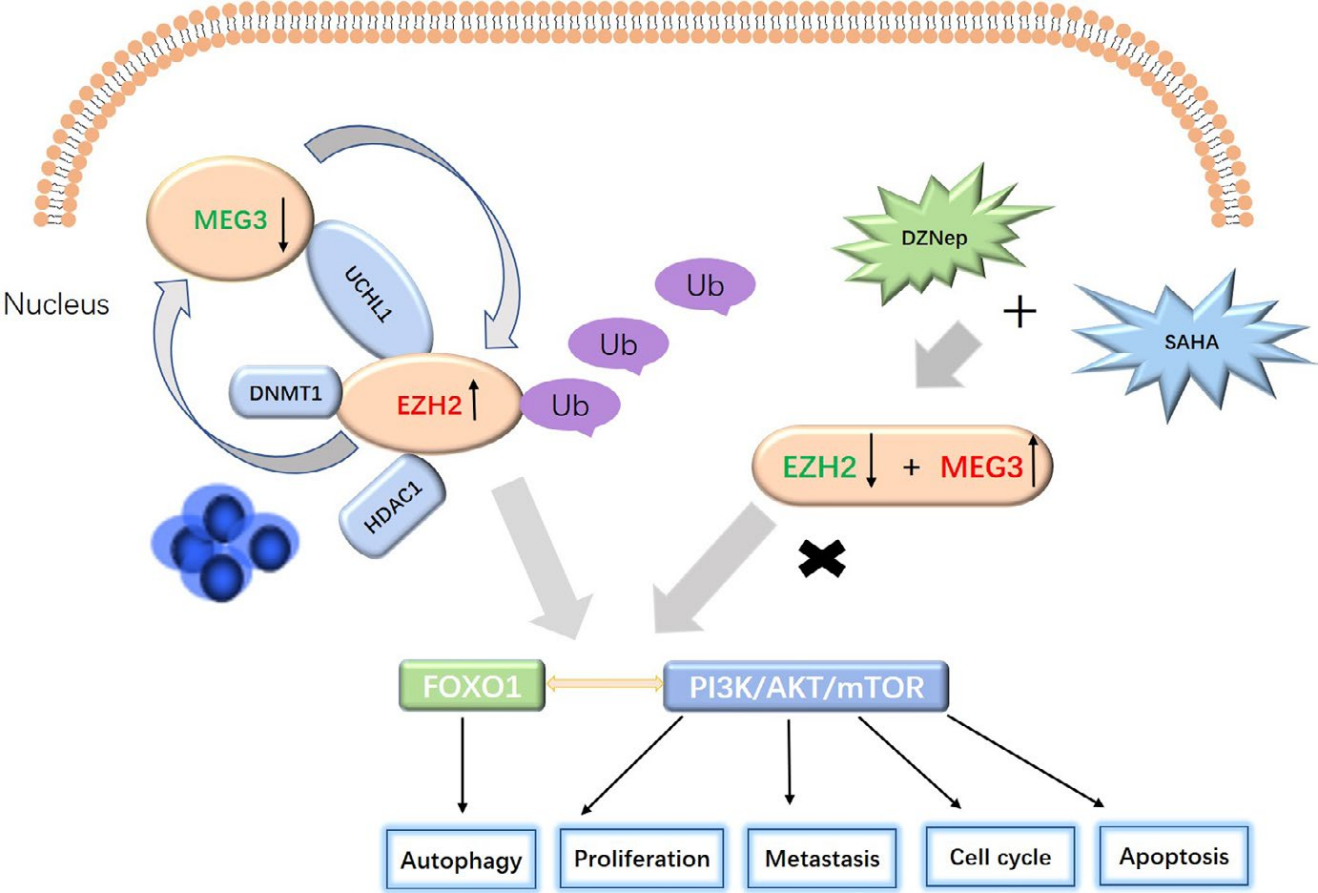
Molecular mechanism diagram of MEG3 and EZH2 in NB

Compared with control and single‐drug treatment groups, co‐treatment with DZNep and SAHA induced a more significant decrease in *EZH2*, *DNMT1* and *HDAC1* expression (Figure 7A). Furthermore, DZNep and SAHA together inhibited the *PI3K*/*AKT*/*mTOR* and *FOXO1* pathways to a greater extent than each agent alone (Figure 7B). CCK‐8 and EdU assays revealed that both DZNep and SAHA induced a decrease in cell proliferation, with the combination treatment achieving a greater inhibitory effect (Figure 7C–H). Flow cytometry analysis revealed an increased proportion of apoptosis in the combined treatment group (Figure S1Q–S). In the colony formation assay, the DZNep‐SAHA combination decreased colony formation ability to a greater extent than DZNep or SAHA alone (Figure S1T). Moreover, data from transwell experiments showed that co‐treatment with DZNep and SAHA suppressed cell migration and invasion to a greater extent than DZNep or SAHA alone (Figure 7I–M). Overall, the combination of DZNep and SAHA not only induced greater inhibition of cell proliferation, migration, invasion and colony formation but also promoted apoptosis through inactivating the *PI3K*/*AKT*/*mTOR*/*FOXO1* pathway (Figure 8).

## 4 DISCUSSION

Previous experiments by our group showed that MEG3 inhibits NB autophagy through *FOXO1* and EMT through *mTOR* in vitro. Moreover, MEG3 and *EZH2* function as mutual negative regulators of each other.^24^ Here, we further explored the anti‐tumour effects of MEG3 in vivo and attempted to clarify the specific molecular mechanisms underlying the regulatory loop of MEG3 and *EZH2*.

In the current study, overexpression of MEG3 inhibited NB growth, consistent with previous in vitro results. Furthermore, upregulation of *EZH2* in MEG3‐overexpressing cells decreased the anti‐tumour effect of MEG3. Cell proliferation, colony formation, migration, invasion and autophagy were inhibited, and apoptosis was promoted by MEG3, which were reversed by *EZH2*. Moreover, the autophagy, EMT and *FOXO1*/*mTOR* pathways were rescued by *EZH2*. RNA‐seq and ChIP‐seq findings further suggested that MEG3 inhibits the *PI3K*/*AKT* pathway via *EZH2*, which is upstream of *mTOR*. We identified the SET domain as a key player in *EZH2* activity based on the finding that its depletion led to loss of the rescue effects of *EZH2* against MEG3. Importantly, *UCHL1* was shown to serve as a bridge mediating MEG3 degradation via ubiquitination of *EZH2*. However, the specific mechanisms underlying MEG3‐mediated inhibition of *UCHL1* are currently unknown. Regulation of downstream target genes by *EZH2* relies on both PRC2 and non‐PRC2 pathways. Data from our experiments suggest that *EZH2* regulates *PIK3AP1* and *FCRL5* in a PRC2‐dependent manner. Additionally, 11 genes, including *SLC7A8*, *HS6ST2*, *LATS2*, *GNB4*, *PDIA2*, *DLX4*, *MET*, *SLC1A1*, *FAM66D* and *CAV2*, appear to be regulated by *EZH2* via non‐PRC2‐dependent pathways. However, the issue of whether these downstream genes contribute to NB and the underlying mechanisms remains to be established.

Conversely, *EZH2* interacts with both *DNMT1* and *HDAC1* to induce MEG3 silencing in NB. Since MEG3 and *EZH2* are mutually regulated in NB and expression of MEG3 is regulated by the epigenetic molecules *EZH2*, *DNMT1* and *HDAC1*, expression of these molecules upstream of MEG3 was inhibited with a view to achieving suppression of NB. *EZH2* is a key tumour‐targeting molecule and DZNep is an effective histone methyltransferase inhibitor.^25^, ^26^, ^27^ Histone deacetylase is an enzyme that plays a key role in regulating gene expression by remodelling chromatin structure.^28^ Aberrant histone acetylation caused by an imbalance in expression and activity of *HDACs* is known to promote progression of a variety of tumours, including NB.^29^, ^30^ *HDACs* (in particular, *HDAC1* and *HDAC2*) can interact with the PRC2 complex to cooperatively regulate downstream genes.^31^

In addition to *EZH2*, *DNMTs* are important epigenetic regulators involved in transcriptional inhibition.^32^, ^33^ Among the *DNMTs*, *DNMT1* maintains methylation while *DNMT3A* and *DNMT3B* function in de novo methylation.^34^, ^35^ *DNMTs* modify CpG island cytosine methylation in the promoter region to inhibit gene expression and inhibition of methylation reactivates gene expression.^36^, ^37^ Mutation or overexpression of *DNMTs* is closely associated with the occurrence and development of various cancer types, such as acute myeloid leukaemia, breast cancer and prostate cancer.^38^ Multiple CpG islands with hypermethylation exist in the promoter region which may be related to the low expression of MEG3 in NB. Using GST pulldown experiments, Vire et al. showed that *EZH2* binds *DNMT1*, *DNMT3A* and *DNMT3B*. Additionally, *DNMTs* interact with the PRC2 complex component, *EED*, and activities of endogenous *EED*, *SUZ12* and *DNMTs* are known to be interlinked.^39^ *EZH2* recruits *DNMTs* to induce hypermethylation of CpG islands in the promoter of the target gene, leading to inhibition of its expression.^40^

At present, the development of inhibitors of epigenetic‐related factors as therapeutic agents is under extensive investigation, including *DNMT* inhibitors (such as 5‐Aza), *EZH2* inhibitors (such as DZNep and Tazemetostat) and *HDAC* inhibitors (such as SAHA).^41^ Clinical trials have revealed promising results in terms of anti‐cancer efficacy. Early last year, The United States Food and Drug Administration accelerated the approval of the *EZH2* inhibitor Tazemetostat (developed by Epizyme) for marketing. The indications are mainly for patients with advanced or extensive metastatic epithelioid sarcoma who are unable to undergo radical surgery, which is a landmark in *EZH2* inhibitor development.^42^ The *HDAC* inhibitor, Vorinostat (SAHA, developed by Merck) has not only been approved as a treatment agent for T‐cell lymphoma but is also undergoing phase 2 and 3 clinical trials for breast cancer and non‐small cell lung cancer.^43^ Since inhibitors of *DNMTs*, *EZH2* or *HDACs* are all non‐specific with regulatory effects on numerous genes, combinations of these drugs may effectively enhance efficacy and reduce side effects.^44^ In the present study, we showed that compared with the control and single inhibitor treatment groups, co‐treatment with DZNep and SAHA inhibited *EZH2*, *HDAC1*, *DNMT1* and *PI3K*/*AKT*/*mTOR*/*FOXO1* signalling to a more significant extent. Moreover, the DZNep‐SAHA combination induced greater inhibition of proliferation, migration and invasion of NB in vitro. However, further efficacy and safety experiments are warranted to validate the clinical utility of combination therapy.

In conclusion, MEG3 plays a tumour suppressor role in NB. Concomitant downregulation of MEG3 and upregulation of *EZH2* promotes the occurrence and development of NB. Combined blockage of *EZH2* and *HDAC1* may be effective in treatment of NB cases with low MEG3 and high *EZH2* expression.

## CONFLICT OF INTEREST

The authors have no conflicts of interest to declare.

## AUTHOR CONTRIBUTIONS

**Mujie Ye:** Conceptualization (lead); Data curation (lead); Formal analysis (lead); Investigation (lead); Methodology (lead). **Runnan Gao:** Conceptualization (equal); Data curation (equal); Formal analysis (equal); Investigation (equal); Methodology (equal). **Shiyu Chen:** Conceptualization (equal); Data curation (equal); Formal analysis (equal); Methodology (equal). **Meng Wei:** Conceptualization (equal); Data curation (equal); Formal analysis (supporting); Investigation (equal); Methodology (supporting). **Jing Wang:** Data curation (supporting); Formal analysis (supporting); Investigation (supporting); Methodology (supporting). **Bowen Zhang:** Conceptualization (equal); Data curation (supporting); Formal analysis (supporting); Investigation (supporting); Methodology (supporting). **Suwen Wu:** Data curation (supporting); Formal analysis (equal); Investigation (supporting); Methodology (supporting). **Yuexin Xu:** Conceptualization (supporting); Data curation (supporting); Formal analysis (supporting); Investigation (supporting); Methodology (supporting). **Peixuan Wu:** Conceptualization (supporting); Data curation (supporting); Formal analysis (supporting). **Xin Chen:** Conceptualization (supporting); Data curation (supporting); Formal analysis (supporting). **Jing Ma:** Investigation (equal); Supervision (equal); Writing – review & editing (equal). **Duan Ma:** Conceptualization (equal); Project administration (equal); Supervision (equal); Writing – review & editing (equal). **Kuiran Dong:** Conceptualization (lead); Funding acquisition (lead); Project administration (lead); Resources (lead); Supervision (lead); Writing – review & editing (lead).

## ETHICAL APPROVAL

The studies involving human tissues and animal experiments were reviewed and approved by The Ethics Committee of Childrens’ Hospital of Fudan University. And all of the patients signed informed consent forms before study.

## Supporting information

Supplementary Material

## Data Availability

All data from this study are available from the corresponding author.
