# Supplementary material for: Downregulation of MEG3 and upregulation of EZH2 cooperatively promote neuroblastoma progression
Source: J Cell Mol Med. 2022 Mar 8;26(8):2377–91. doi: 10.1111/jcmm.17258 (PMC8995459; doi:10.1111/jcmm.17258)
Supplement: Supplementary file 1 — Supplementary Material [file JCMM-26-2377-s001.pdf]

SupplementaryTable1 Antibody information

Antibody	Company	Catalogue	Dilution ratio
E-cadherin	CST	3195T	1：1000
N-cadherin	Proteintech	22018-1-AP	1：1000
ZEB1	Proteintech	21544-1-AP	1：1000
Vimentin	Santa Cruz	Sc-6206	1：1000
Snail1	CST	3879S	1：1000
Slug	Santa Cruz	Sc-166476	1：1000
GAPDH	Proteintech	60004-1-Ig	1：5000
β-Actin	Proteintech	60008-1-Ig	1：5000
LC3	CST	12741S	1：1000
Beclin1	CST	3495S	1：1000
ATG3	CST	3415S	1：1000
ATG5	CST	12994S	1：1000
ATG12	CST	4180S	1：1000
mTOR	CST	2983S	1：1000
p-mTOR2481	CST	2974S	1：1000
Raptor	CST	2280S	1：1000
Rictor	CST	2114S	1：1000
GβL	CST	3274S	1：1000
FOXO1	CST	2880S	1：1000
EZH2	CST	5246S	1：1000
PI3K	Proteintech	20584-1-AP	1：1000
AKT	CST	9272S	1：1000
p-AKT ser473	CST	4060T	1：1000
H3	CST	4499S	1：1000
H3K27me3	CST	9733S	1：1000
H3K27ac	CST	8173S	1：1000
DNMT1	Abcam	ab134148	1：1000
DNMT3A	CST	32578S	1：1000
DNMT3B	CST	57868S	1：1000
HDAC1	Abcam	ab109411	1：1000
HDAC2	Abcam	ab32117	1：1000
Goat Anti-Mouse IgG	CWBIO	CW0102S	1：5000
Goat Anti-Rabbit IgG	CWBIO	CW0103S	1：2000

SupplementaryTable2 Clinical information of 60 NB patients

No	Gende	Age	Stage	Risk	MYCN	Metastasis
1	female	2m	1	unknown	-	no
2	male	3m	1	low	-	no
3	female	3m	unknown	unknown	-	no
4	male	7m	4s	middle	-	no
5	female	3y	4	high	+	yes
6	male	4y	4	high	unknown	yes
7	female	3y	4	high	-	yes
8	male	2y	4	high	+	yes
9	male	2y	4	high	-	yes
10	female	2y	4	high	unknown	yes
11	male	3m	4s	middle	-	yes
12	female	5y	4	middle	-	no
13	female	4y	unknown	middle	unknown	no
14	male	2m	2	middle	-	no
15	female	9y	1	low	-	no
16	female	1y	2	middle	-	no
17	male	2y	4	high	unknown	yes
18	male	5y	4	high	-	yes
19	male	4y	4	high	-	yes
20	male	2m	1	middle	+	yes
21	female	3m	unknown	middle	-	no
22	female	2y	4	high	unknown	yes
23	male	2y	2	middle	unknown	yes
24	female	3y	4	high	+	yes
25	female	6y	2	middle	unknown	yes
26	male	3y	unknown	middle	-	no
27	male	11y	unknown	middle	-	no
28	female	10m	1	low	unknown	no
29	female	1y	4	high	-	yes
30	male	2y	4	high	-	yes
31	female	6m	4	middle	+	yes
32	female	6y	4	high	unknown	yes
33	female	3y	1	unknown	-	no
34	female	3y	4	high	-	yes
35	male	3y	3	high	+	no
36	male	4y	4	high	-	yes
37	female	6m	1	unknown	unknown	no
38	male	5y	4	high	-	yes
39	female	3y	4	high	+	yes
40	female	2y	unknown	middle	unknown	no
41	male	5y	unknown	unknow	unknown	no

42	male	2m	3	middle	-	no
43	female	4y	1	low	-	no
44	male	4y	4	high	+	yes
45	female	4y	4	high	-	yes
46	male	9y	4	high	unknown	yes
47	male	1y	4	high	unknown	no
48	female	8y	4	high	unknown	yes
49	female	2m	unknown	unknown	unknown	no
50	male	3y	3	high	+	yes
51	male	7m	4s	middle	-	no
52	female	5y	4	unknown	unknown	no
53	male	2y	4	high	+	yes
54	female	4y	3	high	-	yes
55	male	5y	4	high	unknown	yes
56	male	1y	4	middle	-	yes
57	female	1y	4	high	-	yes
58	male	1y	3	high	+	yes
59	female	4y	4	high	-	yes
60	male	2y	2	high	unknown	yes

Supplementary Figure 1 A, B, Overexpression of EZH2, but not ΔSET EZH2, rescues apoptosis induced by MEG3 in SK-N-BE(2)C cells; C, catRAPID omics revealed the potential binding site of MEG3 and UCHL1; D, Co-localization of EZH2 and UCHL1 in SK-N-BE(2)C and SK-N-AS cells, magnification is 100×; E, F, IC_{50} of DZNep and SAHA in SK-N-BE(2)C cells; G, Q-PCR for detection of knockdown efficiency of DNMT1, DNMT3A and DNMT3B; H, I, QPCR for detection of knockdown efficiency of HDAC1 and HDAC2; J, K, L, Western blot for detection of knockdown efficiency of DNMT1, DNMT3A, and DNMT3B, respectively; M, N, Western blot for detection of knockdown efficiency of HDAC1 and HDAC2; O, Western blot showing successful overexpression of DNMT1; P, Western blot showing successful overexpression of HDAC1; Q, R, S, Flow cytometry for detection of apoptosis in SK-N-AS and SK-N-BE(2)C cells after DZNep (10 μm) and SAHA (10 μm) treatment for 48 h, respectively; T, Examination of colony formation ability of SK-N-AS and SK-N-BE(2)C cells treated with DZNep (10 μm) and SAHA (10 μm) for 48 h, respectively.

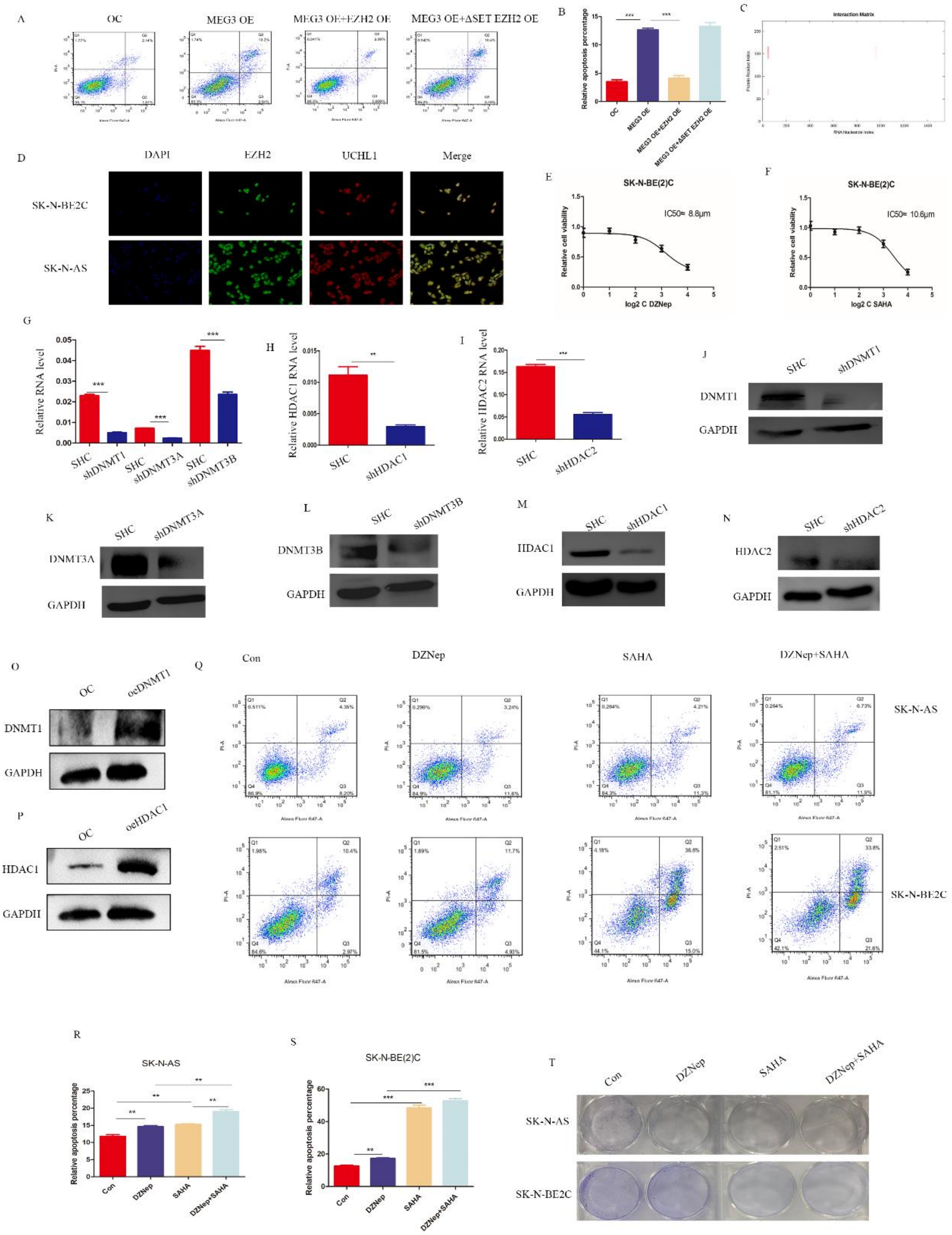

A
OC
MEG3 OE
MEG3 OE+EZH2 OE
MEG3 OE+ΔSET EZH2 OE
B
Relative apoptosis percentage
OC
MEG3 OE
MEG3 OE+EZH2 OE
MEG3 OE+ΔSET EZH2 OE
C
Interaction Matrix
D
DAPI
EZH2
UCHL1
Merge
SK-N-BE2C
SK-N-AS
E
SK-N-BE(2)C
IC50= 8.8μm
Relative cell viability
log2 C DZNep
F
SK-N-BE(2)C
IC50= 10.6μm
Relative cell viability
log2 C SAHA
G
Relative RNA level
SHC
shDNMT1
SHC
shDNMT3A
SHC
shDNMT3B
H
Relative HDAC1 RNA level
SHC
shHDAC1
I
Relative HDAC2 RNA level
SHC
shHDAC2
J
SHC
shDNMT1
DNMT1
GAPDH
K
SHC
shDNMT3A
DNMT3A
GAPDH
L
SHC
shDNMT3B
DNMT3B
GAPDH
M
SHC
shHDAC1
HDAC1
GAPDH
N
SHC
shHDAC2
HDAC2
GAPDH
O
OC
oeDNMT1
DNMT1
GAPDH
P
OC
oeHDAC1
HDAC1
GAPDH
Q
Con
DZNep
SAHA
DZNep+SAHA
SK-N-AS
SK-N-BE2C
R
SK-N-AS
Relative apoptosis percentage
Con
DZNep
SAHA
DZNep+SAHA
S
SK-N-BE(2)C
Relative apoptosis percentage
Con
DZNep
SAHA
DZNep+SAHA
T
Con
DZNep
SAHA
DZNep+SAHA
SK-N-AS
SK-N-BE2C
